# Alten- und Pflegeheime – die COVID-19-Pandemie als Mahnung: Infektionshygienische Maßnahmen und Einflussfaktoren auf die Gesundheit der Bewohnenden

**DOI:** 10.1007/s00103-023-03657-9

**Published:** 2023-02-07

**Authors:** Dunja Said, Muna Abu Sin, Arina Zanuzdana, Birgitta Schweickert, Tim Eckmanns

**Affiliations:** 1 Fachgebiet 37 – Nosokomiale Infektionen, Surveillance von Antibiotikaresistenz und -verbrauch, Robert Koch-Institut, Seestr. 10, 13353 Berlin, Deutschland; 2 Institut für Hygiene und Umweltmedizin, Campus Benjamin Franklin, Charité – Universitätsmedizin Berlin, Berlin, Deutschland

**Keywords:** SARS-CoV‑2, Nichtpharmakologische Maßnahmen, Surveillance, Impfungen, Ausbrüche, SARS-CoV‑2, Nonpharmaceutical interventions, Surveillance, Vaccinations, Outbreaks

## Abstract

Die COVID-19-Pandemie hat die Vulnerabilität der Alten- und Pflegeheimbewohnenden aufgrund ihres erhöhten Risikos für einen schwerwiegenden oder tödlichen COVID-19-Verlauf verdeutlicht. Um die Bewohnenden in den Einrichtungen in Anbetracht hoher Inzidenzen von severe acute respiratory syndrome coronavirus type 2 (SARS-CoV-2) in der Gesamtbevölkerung zu schützen, wurde eine Reihe von Infektionsschutzmaßnahmen empfohlen, die im Verlauf der Pandemie zu einem Rückgang der COVID-19-Fälle und -Todesfälle in den Einrichtungen geführt haben. Gleichzeitig hat sich jedoch gezeigt, dass in Alten- und Pflegeheimen häufig einige Faktoren existieren, welche die Umsetzung von Infektionsschutzmaßnahmen erschweren und einen erheblichen Einfluss auf die Gesundheit der Bewohnenden ausüben.

Herausforderungen ergeben sich vor allem durch die Arbeitsbedingungen (Mangel an Personal grundsätzlich und mit entsprechenden Qualifikationen, arbeitsbedingte Belastungen), durch die Versorgung der Bewohnenden (medizinisch und psychosozial) sowie durch strukturelle und einrichtungsspezifische Faktoren (u. a. Größe von Heimen).

Lösungskonzepte für diese Probleme zeigen, dass die Umsetzung von Infektionsschutzmaßnahmen nicht für sich alleine steht, sondern als Teil eines Konzeptes zur Neugestaltung der Arbeits‑, Wohn- und Lebensbereiche der Beschäftigten und der Bewohnenden der Einrichtungen betrachtet werden sollte. Dabei gilt es, den Infektionsschutz in Alten- und Pflegeheimen nicht ausschließlich in Hinblick auf zukünftige Pandemien zu planen, sondern dessen Relevanz auch für bereits jetzt bestehende Gesundheitsgefahren, wie nosokomiale Infektionen, Antibiotikaresistenzen oder Influenza, zu beachten.

## Abstract (en)

The COVID-19 pandemic once again highlighted the vulnerability of residents of long-term care facilities due to their increased risk of a severe or fatal course of COVID-19. To protect the residents in the face of high incidences of severe acute respiratory syndrome coronavirus 2 (SARS-CoV‑2) in the general population, a number of measures for infection protection were recommended, which led to a decrease of COVID-19 case numbers and deaths in the facilities during the pandemic. However, factors in the facilities that hampered the implementation of these measures and represented a significant impact on the health of the residents also became visible.

The challenges concern working conditions (e.g., lack of staff, both in general and with corresponding competencies, and work-related stress), the care of residents (e.g., medical and psychosocial), and structural as well as facility-related factors (e.g., facility size).

Approaches to these problems indicate that a successful implementation of infection control measures should be part of a concept for redesigning the work and living conditions of the staff and residents of the facilities. It is important to note that infection control should not be planned exclusively with regard to future pandemics, but is of relevance for health risks that already exist in the facilities, such as healthcare-associated infections, antibiotic resistances, or influenza.

## Einleitung

Die Steigerung der Lebenserwartung und der damit wachsende Anteil an Personen hohen Alters hat in den letzten Jahren in Deutschland zu einem zunehmenden Anteil an pflegebedürftigen Menschen geführt [1]. Nach Angaben des statistischen Bundesamtes beträgt die Anzahl der Pflegebedürftigen derzeit ca. 4,1 Mio., wobei mit rund 80 % der überwiegende Anteil zu Hause, entweder durch Angehörige (51,3 %) oder ambulante Pflege- und Betreuungsdienste (23,8 %) bzw. eine Kombination von beidem, versorgt wird, während die anderen 20 % vollstationär in einem der 15.000 Alten- und Pflegeheime untergebracht sind [1, 2].

Insbesondere die Bewohnenden dieser Einrichtungen gehören zu den Hochrisikogruppen für einen schweren oder tödlichen Verlauf von Infektionskrankheiten wie COVID-19. Der Vulnerabilität liegen verschiedene Faktoren zugrunde, wie Gebrechlichkeit und Immunschwäche sowie Komorbiditäten, insbesondere Herz-Kreislauf-Erkrankungen, Diabetes oder Demenz. In Altenheimen leben darüber hinaus auch besonders häufig hochaltrige Personen ab 80 Jahren, welche z. B. in Kombination mit einer Demenzerkrankung 2 wesentliche Risikofaktoren für einen tödlichen COVID-19-Verlauf vereinen [3].

Neben diesen individuellen Risikofaktoren existiert auch eine Reihe einrichtungsspezifischer Bedingungen, welche zu einem erhöhten Erkrankungs- und Sterberisiko der Bewohnenden beitragen. In den Einrichtungen kommen die dort lebenden und arbeitenden Menschen auf verhältnismäßig engem Raum und mit geringem Abstand regelmäßig zusammen. Gemeinsame Aktivitäten und Interaktionen der Bewohnenden untereinander, welche neben den Gemeinschaftsräumlichkeiten häufig auch Wohn- und Sanitärbereiche miteinander teilen [4], und der enge Kontakt zwischen den Bewohnenden und den sie pflegenden Beschäftigten stellen ein erhöhtes Übertragungsrisiko dar [5]. Eingetragen werden die Infektionen dabei auf unterschiedlichen Wegen. Ein besonderes Risiko geht dabei von den Beschäftigten aus, welche z. B. aufgrund einer unerkannten asymptomatischen Erkrankung bei der Arbeit erscheinen und auch häufig in unterschiedlichen Einrichtungen tätig sind [6, 7].

In Hinblick auf die besondere gesundheitliche Gefährdungslage der Bewohnenden von Alten- und Pflegeheimen widmet sich dieser Artikel den eingesetzten Infektionsschutzmaßnahmen und setzt diese in den Kontext bestehender Herausforderungen, welche sich in Bezug auf die Personalsituation, die Versorgung der Bewohnenden und die strukturellen sowie einrichtungsspezifischen Faktoren ergeben. Dabei ergibt sich die Relevanz dieser Thematik nicht ausschließlich in Hinblick auf die jetzige COVID-19-Pandemie oder zukünftige Pandemien, sondern auch aus dem Umgang mit bereits seit längerer Zeit bestehenden Gesundheitsgefahren für die Bewohnenden der Einrichtungen, wie Antibiotikaresistenzen oder Influenza.

## Infektionsepidemiologische und infektionshygienische Lage in Alten- und Pflegeheimen seit Beginn der COVID-19-Pandemie

Mit Beginn der COVID-19-Pandemie wurde auch in Deutschland schnell sichtbar, dass Personen ab 60 Jahren deutlich häufiger von COVID-19-bedingten Hospitalisierungen und Tod betroffen sind als Menschen jüngerer Altersgruppen [8]. Dabei ist das Risiko, an COVID-19 zu versterben, für ältere Personen, die zugleich auch Bewohnende eines Alten- und Pflegeheims sind, nochmal höher als für Personen gleichen Alters, die nicht in einer solchen Einrichtung untergebracht sind [9]. Deutlich wurde dies insbesondere während der ersten beiden Pandemiewellen. So offenbarten Meldedatenanalysen für den Zeitraum 2020 bis Anfang 2021, dass nur 5 % der Personen über 65 Jahren in Deutschland in einem Alten‑/Pflegeheim untergebracht sind, aber diese Gruppe zugleich rund 21 % der COVID-19-Fälle über 65 Jahre und fast 30 % aller COVID-19-Todesfälle über 65 Jahre stellt [9].

Bei Betrachtung der COVID-19-Todesfälle bei Heimbewohnenden unter allen COVID-19-Todesfällen in der Gesamtbevölkerung scheint Deutschland im europäischen Vergleich allerdings noch verhältnismäßig gut dazustehen: So betrug diesbezüglich mit Stand Mai 2022 der Anteil in Deutschland 29 % und war somit deutlich niedriger als beispielsweise in den Niederlanden (52 %) oder Schweden (94 %; [10]).

Eine wesentliche Verbesserung der Lage in den Einrichtungen stellte sich mit Einführung der COVID-19-Impfungen ein. So bestand insbesondere während der ersten beiden Pandemiewellen eine starke Assoziation zwischen Ausbrüchen in Alten- und Pflegeheimen und der Inzidenz von severe acute respiratory syndrome coronavirus type 2 (SARS-CoV-2) in der Allgemeinbevölkerung, welche erst durch die Kombination aus Impfungen und weiteren Maßnahmen zu großen Teilen entkoppelt werden konnte [9, 11].

Ein Vergleich des Zeitraums vor (März bis Dezember 2020) und nach Beginn der Impfkampagne (April bis Dezember 2021) in Deutschland zeigte außerdem, dass zwischen den beiden Zeiträumen in den Einrichtungen die mediane Anzahl an COVID-19-Fällen pro Ausbruch von 21 auf 8 fiel und auch bei den verstorbenen Fällen unter den Bewohnenden ein Rückgang von 21,1 % auf 13,6 % zu verzeichnen war [12].

Trotz dieser positiven Entwicklungen waren seit August 2021 wieder zunehmend Ausbrüche in den Einrichtungen zu beobachten [13]. Ein wesentlicher Grund hierfür liegt an der anhaltend hohen und zwischenzeitlich auch stark ansteigenden SARS-CoV-2-Inzidenz in der Allgemeinbevölkerung, welche seit Anfang 2022 mit der Verbreitung der SARS-CoV-2-Variante Omikron in Deutschland neue Höchstwerte erreichte und sich auch über die darauffolgenden Sommermonate im Vergleich zu den 2 Vorsommerjahren auf einem hohen Niveau hielt [14].

### Infektionsschutzmaßnahmen

Um die Bewohnenden der Einrichtungen vor SARS-CoV-2-Infektionen und daraus resultierenden Todesfällen bestmöglich zu schützen, wurden sowohl in Deutschland als auch international zu Beginn der Pandemie Empfehlungen zur Infektionsprävention und zum Infektionsmanagement in den Einrichtungen veröffentlicht [15, 16]. Auch wenn ein Teil der Maßnahmen im Laufe der Pandemie entschärft oder gänzlich aufgehoben wurde, sind diese nach wie vor von Relevanz und werden entsprechend der infektionsepidemiologischen Entwicklung stetig angepasst und überarbeitet [15]. Sie beinhalten im Wesentlichen die im Folgenden dargestellten Maßnahmen, welche in umfassenderer Form in dem Dokument* Prävention und Management von COVID-19 in Alten- und Pflegeeinrichtungen und Einrichtungen für Menschen mit Beeinträchtigungen und Behinderungen *des Robert Koch-Instituts (RKI; [15]) zu finden sind.

#### Hygiene- und Infektionskontrollmaßnahmen

Eine wesentliche Voraussetzung, um den Eintrag und die Verbreitung von SARS-CoV-2-Infektionen in die Einrichtungen zu vermeiden, sind das Vorhandensein und die konsequente Umsetzung eines Hygienekonzeptes, welches die entsprechenden Hygiene- und Infektionskontrollmaßnahmen beinhaltet. Dieses sollte neben Händehygiene und Basishygienemaßnahmen (u. a. Abstandsregelung, Tragen medizinischer Masken durch Beschäftigte und Besuchende sowie regelmäßiges Lüften der Innenräume) auch erweiterte Maßnahmen beinhalten (z. B. Nutzung persönlicher Schutzausrüstungen, Desinfektion und Reinigung) sowie, ggf. in Abhängigkeit von landesrechtlichen Vorgaben und der epidemiologischen Lage, Regelungen zu Besuchsmöglichkeiten und Testungen [15].

Umfassende Maßnahmen zur Hygiene und Infektionsprävention in den Einrichtungen sind jedoch nicht alleine im Kontext von COVID-19 von Bedeutung. Relevanz besteht diesbezüglich z. B. auch in Hinblick auf die Gefährdung durch multiresistente Erreger, welche ebenfalls eine akute Gesundheitsgefährdung für die Bewohnenden darstellen [17]. Die *Kommission für Krankenhaushygiene und Infektionsprävention *(KRINKO) beim RKI hat im Zusammenhang mit den besonderen Infektionsgefahren im Setting von Pflegeeinrichtungen bereits vor einigen Jahren Empfehlungen veröffentlicht (derzeit in Überarbeitung), in denen diesbezüglich relevante Maßnahmen umfangreich dargelegt sind [18].

#### Kontaktpersonennachverfolgung und Fallmanagement

Neben der Prävention umfasst Infektionsschutz in den Einrichtungen insbesondere die Verhinderung weiterer Infektionen, falls diese in die Einrichtung getragen wurden. Die Nachverfolgung von COVID-19-Kontaktpersonen sowie das Fallmanagement wurden schon zu Beginn der Pandemie als wichtige Containment-Maßnahmen für die Allgemeinbevölkerung identifiziert und auch für die Einrichtungen empfohlen [15].

Ziel dabei ist, Infektionen möglichst schnell zu erkennen, um somit eine angemessene medizinische Versorgung der erkrankten Person gewährleisten zu können und größere Ausbrüche in den Einrichtungen zu vermeiden. Hierfür bedarf es der Anwendung entsprechender Quarantäne- und Isolierungsmaßnahmen sowohl unter den Bewohnenden als auch unter den Beschäftigten, wobei die konkrete Umsetzung der Situation angepasst erfolgen sollte [15]. Laut den Ergebnissen eines Rapid-Reviews stellen die frühzeitige Identifizierung und Isolierung von COVID-19-Fällen das wichtigste Kontrollinstrument zur Ausbruchseindämmung dar [5].

#### Surveillance- und Monitoring-Systeme

Die Wirkung der oben genannten Maßnahmen ist umso effektiver, je früher die Infektionen identifiziert werden. Dafür bedarf es einer einrichtungsbasierten Surveillance und Kenntnisse der Symptome einer COVID-19-Erkrankung, über die die Bewohnenden und die Beschäftigten der Einrichtungen umfassend aufgeklärt werden sollten [15].

Die Bedeutung einer übergeordneten einrichtungsbasierten Surveillance und von Monitoring-Systemen zeigt sich auch, wenn die auf diese Weise gesammelten Daten generelle Einblicke zur Lage in den Einrichtungen liefern. Beispiele hierfür sind das *Monitoring von COVID-19 und der Impfsituation in Langzeitpflegeeinrichtungen* [19] und die darauffolgende Meldepflicht gemäß § 35 Abs. 6 Infektionsschutzgesetz (IfSG) zur Übermittlung der Impfquoten in den Einrichtungen. Solche Systeme können dazu beitragen, kurzfristig und schnell Erkenntnisse über die Lage in den Einrichtungen zu erhalten und somit zu möglichen akuten Handlungsbedarfen in den Einrichtungen Informationsgrundlagen zur Verfügung zu stellen. Dies unterstreicht, dass Surveillance- und Monitoring-Systeme insbesondere in Bezug auf nosokomiale Infektionen stärker in den Einrichtungen etabliert werden sollten [20].

#### SARS-CoV-2-Testungen

Zahlreiche Studienergebnisse belegen die Bedeutung von Testungen auf SARS-CoV‑2 als Infektionsschutz-Instrument, insbesondere in Hinblick auf Antigen-Schnelltests zur Detektion asymptomatischer Infektionen im Rahmen von regelmäßigen Reihentestungen [5, 6, 21]. Dabei gilt es zu beachten, dass bei symptomatischen Personen PCR-Testungen vorgenommen werden sollten [15]. Weitere wichtige Anforderungen zum sinnvollen und effektiven Einsatz der unterschiedlichen Testarten umfassen z. B. die ausschließliche Nutzung von zertifizierten Tests oder die PCR-Nachtestung nach positivem Schnelltestergebnis [15]. Die einrichtungsspezifischen Testkonzepte stehen dabei in Abhängigkeit der jeweiligen epidemiologischen Lage sowie landesspezifischer Regelungen und sollten im Rahmen der Möglichkeiten mit Unterstützung des Gesundheitsamtes erarbeitet werden [15].

#### Ausbruchsmanagement

Auch ein effektives Ausbruchsmanagement ist beim Auftreten eines COVID-19-Falles in einer Einrichtung essenziell, um die Ausbreitung weiterer Infektionen und das Auftreten von Todesfällen zu verhindern. Die Maßnahmen eines solchen Ausbruchsmanagements umfassen dabei die bereits genannten Interventionen, wie z. B. die Kontaktpersonennachverfolgung, die Identifizierung weiterer Fälle sowie, je nach Ausmaß des Ausbruchs, vorübergehende Einschränkungen hinsichtlich der Besuchsmöglichkeiten [15]. Dabei ist es elementar, dass die Maßnahmen rechtzeitig und schnell umgesetzt werden.

#### Impfungen

Der Rückgang an COVID-19-Fällen, Todesfällen und Ausbrüchen in Alten- und Pflegeheimen verdeutlicht die Bedeutung der Impfungen als Infektionsschutzmaßnahme, welche Ende 2020 mit Beginn der Impfkampagne in Deutschland auf Grundlage der Empfehlung der *Ständigen Impfkommission* (STIKO) prioritär den Bewohnenden und Beschäftigten der Einrichtungen zur Verfügung gestellt wurden. Mit dem Auftreten neuer SARS-CoV-2-Varianten sowie des im Laufe der Zeit sinkenden Impfschutzes zeigte sich jedoch auch die Notwendigkeit, die impfbedingte Schutzwirkung wieder aufzufrischen, weswegen die STIKO den Bewohnenden und Beschäftigten von Pflegeeinrichtungen im Oktober 2021 bzw. Februar 2022 die erste bzw. zweite Auffrischimpfung empfahl. Um bestehende Impflücken zu schließen, wurde darüber hinaus im Dezember 2021 die seit März 2022 in Kraft getretene einrichtungsbezogene Impfpflicht für Beschäftigte in Pflegeeinrichtungen beschlossen [22].

Aktuelle Daten zum Stand der Impfquoten in den Einrichtungen im November 2022 zeigen, dass unter den Bewohnenden und Beschäftigten inzwischen der überwiegende Anteil der Personen mindestens zweimal (jeweils 94 %) bzw. mindestens dreimal (87 % bzw. 76 %) geimpft ist [23]. Gleichzeitig ist jedoch sichtbar, dass in Bezug auf die zweite Auffrischimpfung (Bewohnende bzw. Beschäftigte mit mindestens 4 Impfungen: 52 % bzw. 12 %) weiterhin Impflücken bestehen [23].

### Einflussfaktoren auf die Gesundheit von Alten- und Pflegeheimbewohnenden und Herausforderungen für den Infektionsschutz in den Einrichtungen

**Figure 1:**
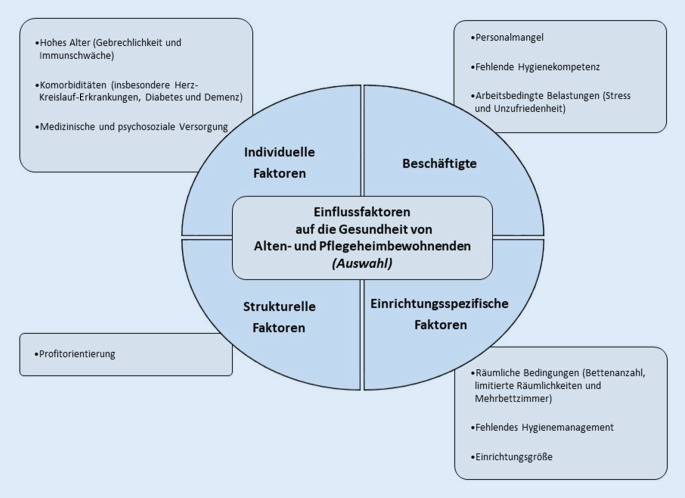

Es gibt eine Reihe an unterschiedlichen Faktoren, welche die Gesundheit von Alten- und Pflegeheimbewohnenden beeinflussen und häufig auch die Umsetzung der oben genannten Maßnahmen in den Einrichtungen erschweren (Abb. 1).

Zugleich existieren aber auch Lösungskonzepte, Empfehlungen und Forderungen, wie diesen Herausforderungen begegnet werden kann. Diese zeigen, dass beim Einsatz von Infektionsschutzmaßnahmen auch psychosoziale und anderweitige Faktoren berücksichtigt werden sollten [15].

#### Personalmangel, fachliche Qualifikation und arbeitsbedingte Belastungen

Eine wesentliche Problematik, welche sich auf den Infektionsschutz in den Einrichtungen auswirkt, ist der bereits seit einigen Jahren bestehende Mangel an fachlich qualifiziertem Personal, sowohl generell als auch in Bezug auf infektionshygienisch geschulte Beschäftigte, welcher sich im Zuge der COVID-19-Pandemie zusätzlich verstärkt hat [24–26]. Die Auswirkungen dieses Mangels zeigen Studienergebnisse, welche darauf hindeuten, dass Einrichtungen mit einem hohen Anteil an qualifizierten Pflegekräften seltener von COVID-19-Fällen betroffen sind als Einrichtungen mit Personalmangel [5, 27].

Deutlich wird dies auch bei einem Vergleich der Größe von COVID-19-Ausbrüchen in Krankenhäusern und Alten- und Pflegeheimen: So schwankte in Deutschland während der ersten 4 COVID-19-Wellen zwischen März 2020 und September 2021 die mediane Ausbruchsgröße in Krankenhäusern zwischen 3 und 5 Fällen, in den Einrichtungen jedoch zwischen 7 und 21 [11]. Die Situation zeigt, dass es neben einer grundsätzlichen Erhöhung des Personals auch der Förderung der Hygienekompetenzen der Beschäftigten bedarf.

Forderungen und mögliche Lösungskonzepte diesbezüglich bestehen bereits und umfassen neben der Notwendigkeit einer angemessenen Vergütung für die Beschäftigten unter anderem ein gesetzlich verpflichtendes Hygienemanagement, die Einstellung hygienebeauftragter Pflegekräfte, die Aufnahme des Faches Hygiene in die Ausbildung zur Pflegefachkraft sowie an die Pflege im Krankenhaus angelehnte höherqualifizierende Ausbildungen und Weiterbildungsmöglichkeiten [24, 28]. Mit Änderung des IfSG im September 2022 wurde bereits die gesetzliche Grundlage dafür geschaffen, die Einrichtungen beim Einsatz von Beauftragten für die Bereiche Impfung, Hygiene und Medikation zu unterstützen [29].

Wie wichtig diese Maßnahmen sind, zeigt sich auch eindrücklich anhand von Untersuchungen zu den Auswirkungen der COVID-19-Lage auf die Arbeitssituation der Beschäftigten. So gab eine überwiegende Mehrheit (94 %) der Beschäftigten von Pflegeeinrichtungen in einer in Deutschland durchgeführten Erhebung an, seit Beginn der COVID-19-Pandemie einer erhöhten Arbeitsbelastung ausgesetzt zu sein, wobei ein großer Teil (40 %) in diesem Zusammenhang auch von Unzufriedenheit mit dem COVID-19-Management ihrer Einrichtungsleitung berichtete [30].

Eindrückliche Schilderungen der pandemiebedingten Belastung zeigten sich dabei auch im Rahmen einer qualitativen Untersuchung, in der von einer „stillen Triage“ berichtet wird und nach der insbesondere während der zweiten Pandemiewelle erkrankte Bewohnende nicht ins Krankenhaus verlegt wurden, zugleich in den Einrichtungen selbst allerdings nicht die Voraussetzungen für eine angemessene medizinische Versorgung vorlagen [31].

#### Medizinische und psychosoziale Versorgung der Bewohnenden

Bis zum Start der Impfkampagne waren Alten- und Pflegeheime bereits mehrere Monate von den Folgen der COVID-19-Pandemie beeinflusst, weswegen die über den Infektionsschutz hinausgehenden direkten und indirekten Auswirkungen auf die Bewohnenden bis zu diesem Zeitpunkt schon deutlich in Erscheinung traten. Neben der generellen Sorge vor einer Infektion ergaben sich weitere Belastungen durch die Besuchsverbote von Familienangehörigen und Bekannten sowie durch die Einschränkungen bzw. den Wegfall sozialer Aktivitäten [31]. Berichtet wurde vor diesem Hintergrund von verstärkten Gefühlen der Isolation, Macht- oder Hilflosigkeit unter den Bewohnenden sowie von Angstzuständen, Depressionen, aggressivem Verhalten und kognitiven Einschränkungen [31, 32].

Darüber hinaus zeigten manche der Bewohnenden Schwierigkeiten bei der Einhaltung der Hygienemaßnahmen [33]. Die Studienlage deutet dabei zumindest in Teilen darauf hin, dass hiervon im besonderen Maße Bewohnende mit Demenzerkrankungen betroffen waren, welche im Falle von Hospitalisierungen durch die Notwendigkeit des Umgebungswechsels und einer intensiveren medizinischen Betreuung durch fremdes Personal einer verstärkten Belastungssituation ausgesetzt waren [34].

Alten- und Pflegeheime stellen ein besonderes Setting dar: Sie umfassen zwar eine pflegerische Betreuung, gleichzeitig handelt es sich bei ihnen jedoch auch um Wohnunterkünfte, in denen sich in der Regel alle relevanten Lebensbereiche und Aktivitäten der Bewohnenden abspielen (Wohnen, Essen, sozialer Austausch usw.), was bei der Umsetzung von infektionshygienischen Maßnahmen unbedingt beachtet werden sollte.

Insbesondere die Tatsache, dass zu Beginn der Pandemie Bewohnende aufgrund der Beschränkungen oftmals während des Sterbeprozesses nicht von Angehörigen begleitet werden konnten, unterstreicht das Ausmaß der Auswirkungen, welche die Infektionsschutzmaßnahmen auf die Lebensumstände der beteiligten Personengruppen hatten. Anhand dessen zeigt sich deutlich das Dilemma der Abwägung zwischen der Notwendigkeit, die Bewohnenden bestmöglich vor einer SARS-CoV-2-Infektion zu schützen, als auch soweit wie möglich vor den gravierenden negativen Folgen der Schutzmaßnahmen selbst. Dies setzt eine adäquate Anpassung der oben genannten Maßnahmen durch die beteiligten Akteur:innen und Behörden an die jeweiligen Gegebenheiten vor Ort und die entsprechende Situation selbst voraus, wobei das Prinzip der Verhältnismäßigkeit dabei eine zentrale Rolle einnimmt [35].

Die S1-Leitlinie zur *Sozialen Teilhabe und Lebensqualität in der stationären Altenhilfe unter den Bedingungen der COVID-19-Pandemie* greift dies auf, in dem hier eine Reihe an Empfehlungen der damit einhergehenden notwendigen Abwägungsprozesse beleuchtet wird. Ein wesentlicher Aspekt in diesem Zusammenhang ist die Umsetzung notwendiger Infektionsschutzmaßnahmen unter Bewahrung der menschlichen Würde, was unter anderem eine sorgfältige Eruierung vorhandener Handlungsoptionen, eine Kommunikation mit den beteiligten Personengruppen sowie eine weitestgehende Aufrechterhaltung der Möglichkeiten zur Bewegungsfreiheit und des sozialen Austausches umfasst [35].

In Bezug auf das psychische Wohlergehen der Bewohnenden werden in der Leitlinie auch die große Bedeutung und der Beitrag der Angehörigen zur Lebensqualität der Bewohnenden in den Mittelpunkt gestellt. Ihnen im Rahmen der jeweils gelten Hygienebestimmungen vor Ort unkomplizierten Zugang zu ermöglichen, sollte unter Berücksichtigung des individuellen Gesundheitszustands der zu besuchenden Personen sowie der strukturellen bzw. räumlichen Gegebenheiten gegeben sein [35].

Die genannten Aspekte zeigen eindrücklich, wie wichtig es ist, infektionshygienische Maßnahmen mit Konzepten der psychosozialen Versorgung, wie einer personzentrierten Pflege der Bewohnenden sowie spezieller Unterstützungs- und Beratungsangebote für die Beschäftigten, zu verbinden [35, 36]. Dies kann zusätzlich auch dazu beitragen, die Compliance der Bewohnenden mit den Maßnahmen zu erhöhen.

Weiteres Potenzial zur Verbesserung der medizinischen und psychosozialen Versorgung der Bewohnenden und somit zu deren Schutz vor Infektionen umfasst die Förderung des Heimärzt:innen-Konzeptes, d. h. von Ärzt:innen, die direkt beim Heim angestellt sind [28, 37], wobei es hier unter Umständen zu Kollisionen mit dem Recht auf freie Ärzt:innenwahl kommen kann.

Eine wichtige Ergänzung in Bezug auf die Versorgung der Bewohnenden stellt daneben das Berufsbild der *Advanced Practice Nurse* dar [38]. Diese spezialisierten Pflegekräfte können andere Beschäftigte der Einrichtungen – insbesondere in Anbetracht von Personalmangel und erhöhter Arbeitsbelastung – dabei unterstützen, zum Teil bestehende Versorgungslücken zu schließen, zu einer qualitativ hochwertigeren Pflege beizutragen und das psychische Wohlbefinden der Bewohnenden zu fördern [39, 40].

#### Strukturelle und einrichtungsspezifische Faktoren

Im Zuge der COVID-19-Pandemie wurde nochmal deutlich, dass strukturelle und einrichtungsspezifische Faktoren in den Einrichtungen existieren, welche die Umsetzung von Infektionsschutzmaßnahmen erschweren können. In Bezug auf die oben genannte Maßnahme der Kohortierung und Isolierung beim Auftreten von COVID-19-Fällen zeigt sich beispielsweise, dass diese aufgrund der räumlichen Bedingungen in manchen Einrichtungen nicht oder nur unzureichend umgesetzt wird bzw. werden kann [4, 24]. Damit übereinstimmend berichten Ergebnisse von internationalen Studien auch, dass eine hohe Bettenanzahl, limitierte Räumlichkeiten und Mehrbettzimmer zu einem gesteigerten Infektionsrisiko für Bewohnende beitragen [5, 32].

Gegenstand von Untersuchungen ist darüber hinaus die Größe von Pflegeheimen. Ergebnisse von Studien weisen darauf hin, dass Bewohnende kleinerer Einrichtungen ein geringeres Risiko haben, an COVID-19 zu erkranken oder zu versterben, als Bewohnende großer Alten- und Pflegeheime, da hier eine größere Zahl an Personen in Gemeinschaftsräumlichkeiten aufeinandertrifft [41, 42].

Problematiken in Hinblick auf eine ausreichende personelle Besetzung mit entsprechenden Qualifikationen, welche sich aus einer profitorientierten Ausrichtung von Pflegeheimen ergeben [43], sowie Hinweise über mögliche Assoziationen zwischen Ausmaß an COVID-19-Ausbrüchen und der Anzahl von Todesfällen im Vergleich zu nichtprofitorientierten Einrichtungen [44] bestärken darüber hinaus den Bedarf für weitere Forschung über den möglichen Einfluss einer gewinnorientierten Ausrichtung von Einrichtungen auf den Infektionsschutz.

Die hier genannten Aspekte werfen daher Fragen hinsichtlich der Gestaltung von Lebens- und Wohnräumen in den Einrichtungen und möglicher Alternativen auf [45]. In europäischen Nachbarländern wurden bereits Konsequenzen gezogen: Im österreichischen Burgenland sieht der *Zukunftsplan Pflege* die Gemeinnützigkeit für Pflegeeinrichtungen in den kommenden Jahren bereits als gesetzliche Pflicht vor [46]. In Dänemark existieren inzwischen gar keine Altenheime mehr [47]. Alternative Konzepte zur Betreuung pflegebedürftiger Personen sehen statt der klassischen Einrichtungen kleinere Heime mit max. 12 Zimmern bzw. sogenannten *Green Houses* vor [41]. Solche Optionen bieten Potenzial zur Umgestaltung der Unterbringungsmöglichkeiten und Lebensräume älterer und pflegebedürftiger Menschen, was nicht nur zum Infektionsschutz, sondern auch zu einer Verbesserung der Lebensqualität dieser Personengruppen beitragen könnte [45]. Ob und inwieweit solche Wohnmodelle für alle Pflegegruppen tatsächlich geeignet sind, sollte Gegenstand weiterer Untersuchungen sein.

## Fazit

Die COVID-19-Pandemie hat verdeutlicht, welch hohem Infektions- und Sterberisiko die Bewohnenden von Alten- und Pflegeheimen ausgesetzt sind und wie sehr deren Situation auch in Abhängigkeit zu der epidemiologischen Lage in der Allgemeinbevölkerung steht. Durch Infektionsschutzmaßnahmen, wie Hygienemaßnahmen, Testungen auf SARS-CoV‑2 und Impfungen, konnte im Verlauf der Pandemie eine Vielzahl von Erkrankungen und Todesfällen unter den Bewohnenden verhindert werden. Gleichzeitig wurden jedoch auch einige Herausforderungen hinsichtlich der Umsetzung dieser Maßnahmen in den Einrichtungen sichtbar, welche z. T. direkt durch die Pandemie bedingt sind, teilweise aber bereits Jahre zuvor bestanden und durch diese verstärkt wurden.

Entsprechende Lösungsansätze zeigen, dass ein umfassender Schutz der Bewohnenden vor Infektionen neben konkreten Maßnahmen, wie z. B. neuer Impfkampagnen zur Schließung von Lücken im Bereich der COVID-19-Auffrischimpfungen, auch die Arbeitsbedingungen der Beschäftigten, die Versorgung der Bewohnenden sowie die räumlichen Gegebenheiten in den Einrichtungen berücksichtigen sollte. Eine wichtige Lektion aus der COVID-19-Pandemie ist, sich bei der Planung von Maßnahmen nicht ausschließlich an zukünftigen Pandemien zu orientieren [48], sondern auch anderweitige und bereits bestehende Gesundheitsgefahren in den Fokus zu nehmen.

Weiterhin zeigt sich, dass die Evidenzlage in Bezug auf die jeweiligen Maßnahmen zukünftig weiter gestärkt werden sollte. So wurde im Rahmen eines Rapid-Reviews aus dem Jahr 2021 eine Reihe nichtpharmakologischer Interventionen untersucht, welche zur Prävention von SARS-CoV-2-Infektionen in Langzeitpflegeeinrichtungen eingesetzt wurden [49]. Die Ergebnisse deuten zwar darauf hin, dass ein Großteil der oben genannten Maßnahmen die Anzahl der Infektionen und Todesfälle reduziert, der Grad der Evidenz ist aufgrund der Anzahl der Studien sowie der Studiendesigns jedoch nur begrenzt [49].

In Hinblick auf die weiter steigende Anzahl an älteren und pflegebedürftigen Personen gilt es daher, bestehende Forschungslücken im Bereich des Infektionsschutzes dieses Personenkreises zu schließen. Geeignete Mittel hierfür wären z. B. die Etablierung und Ausweitung von Monitoring- und Surveillance-Systemen sowie die Durchführung von systematischen Reviews und weiteren Studien, welche sich der Wirksamkeit von Infektionsschutzmaßnahmen in den Einrichtungen widmen [49]. Diese können eine wichtige Grundlage dafür schaffen, weitere Informationen zu Faktoren, welche die Gesundheit von Bewohnenden beeinflussen, zu sammeln, zu interpretieren und darauf basierend Handlungsempfehlungen zu formulieren. Zentraler Bestandteil dieser Empfehlungen sollte dabei stets eine angemessene Abwägung zwischen notwendigen Infektionsschutzmaßnahmen auf der einen und möglichen negativen Auswirkungen auf die psychosoziale Gesundheit der Bewohnenden auf der anderen Seite sein.

## References

[1] Statistisches Bundesamt (2022) Gesundheit. Pflege. https://www.destatis.de/DE/Themen/Gesellschaft-Umwelt/Gesundheit/Pflege/_inhalt.html. Zugegriffen: 25. Sept. 2022

[2] Statistisches Bundesamt (2020) Pflegebedürftige nach Versorgungsart, Geschlecht und Pflegegrade 2019. https://www.destatis.de/DE/Themen/Gesellschaft-Umwelt/Gesundheit/Pflege/Tabellen/pflegebeduerftige-pflegestufe.html. Zugegriffen: 30. März 2022

[3] España PP, Bilbao A, García-Gutiérrez S (2021). Predictors of mortality of COVID-19 in the general population and nursing homes. Intern Emerg Med.

[4] Davidson PM, Szanton SL (2020). Nursing homes and COVID-19: we can and should do better. J Clin Nurs.

[5] Dykgraaf SH, Matenge S, Desborough J (2021). Protecting nursing homes and long-term care facilities from COVID-19: a rapid review of international evidence. J Am Med Dir Assoc.

[6] McMichael TM, Currie DW, Clark S (2020). Epidemiology of Covid-19 in a long-term care facility in king county, washington. N Engl J Med.

[7] Yanes-Lane M, Winters N, Fregonese F (2020). Proportion of asymptomatic infection among COVID-19 positive persons and their transmission potential: a systematic review and meta-analysis. PLoS ONE.

[8] Schilling J, Lehfeld A, Schumacher D (2020). RKI COVID-19 Study Group: Krankheitsschwere der ersten COVID-19-Welle in Deutschland basierend auf den Meldungen gemäß Infektionsschutzgesetz. J. Health Monit..

[9] Schweickert B, Klingeberg A, Haller S (2021). COVID-19-Ausbrüche in deutschen Alten- und Pflegeheimen. Epidemiol Bull.

[10] ECDC (2022) Surveillance data from public online national reports on COVID-19 in long-term care facilities. https://www.ecdc.europa.eu/en/all-topics-z/coronavirus/threats-and-outbreaks/covid-19/prevention-and-control/LTCF-data. Zugegriffen: 27. Sept. 2022

[11] Suwono B, Steffen A, Schweickert B (2022). SARS-CoV-2 outbreaks in hospitals and long-term care facilities in Germany: a national observational study. Lancet Reg Health Eur.

[12] Said D, Suwono B, Schweickert B, Schönfeld V, Eckmanns T (2022). SARS-CoV-2 outbreaks in care homes for the elderly and disabled in Germany. A comparative epidemiological analysis of the periods before and after the beginning of the vaccination campaign. Dtsch Arztebl Int.

[13] RKI (2022) Wöchentlicher Lagebericht des RKI zur Coronavirus-Krankheit-2019 (COVID-19). 22.09.2022. https://www.rki.de/DE/Content/InfAZ/N/Neuartiges_Coronavirus/Situationsberichte/Wochenbericht/Wochenbericht_2022-09-22.pdf?__blob=publicationFile. Zugegriffen: 25. Sept. 2022

[14] RKI (2022) Covid-19-Trends in Deutschland im Überblick. https://www.rki.de/DE/Content/InfAZ/N/Neuartiges_Coronavirus/Situationsberichte/COVID-19-Trends/COVID-19-Trends.html?__blob=publicationFile#/home. Zugegriffen: 8. Sept. 2022

[15] RKI (2022) Prävention und Management von COVID-19 in Alten-und Pflegeeinrichtungen und Einrichtungen für Menschen mit Beeinträchtigungen und Behinderungen. https://www.rki.de/DE/Content/InfAZ/N/Neuartiges_Coronavirus/Alten_Pflegeeinrichtung_Empfehlung.pdf?__blob=publicationFile. Zugegriffen: 1. Sept. 2022 (Empfehlungen des Robert Koch-Instituts für Alten- und Pflegeeinrichtungen und Einrichtungen für Menschen mit Beeinträchtigungen und Behinderungen und für den öffentlichen Gesundheitsdienst. V.29, 27.05.2022)

[16] CDC (2022) Interim infection prevention and control recommendations to prevent SARS-coV‑2 spread in nursing homes. Nursing homes & long-term care facilities. https://www.cdc.gov/coronavirus/2019-ncov/hcp/long-term-care.html. Zugegriffen: 14. Aug. 2022

[17] Esposito S, Leone S, Noviello S, Lanniello F, Fiore M (2007). Antibiotic resistance in long-term care facilities. New Microbiol.

[18] RKI (2005). Infektionsprävention in Heimen Empfehlung der Kommission für Krankenhaushygiene und Infektionsprävention beim Robert Koch-Institut (RKI). Bundesgesundheitsbl.

[19] RKI (2022) Monitoring von COVID-19 und der Impfsituation in stationären Langzeitpflegeeinrichtungen und in ambulanten Pflege- und Betreuungsdiensten. https://www.rki.de/DE/Content/Infekt/Impfen/ImpfungenAZ/COVID-19/Monitoring_Covid-19_Impfen_Langzeitpflege.html. Zugegriffen: 25. Sept. 2022

[20] Schmidt N, Marujo V, Eckmanns T, Zacher B, Arvand M, Ruscher C (2022). Nosokomiale Infektionen und Antibiotikaanwendung in Langzeitpflegeeinrichtungen. Deutsche Ergebnisse der dritten europäischen Punkt-Prävalenz-Erhebung HALT-3. Bundesgesundheitsbl.

[21] Kim JJ, Coffey KC, Morgan DJ, Roghmann MC (2020). Lessons learned—outbreaks of COVID-19 in nursing homes. Am J Infect Control.

[22] Bundestag (2021) Impfpflicht für Gesundheits- und Pflegepersonal ab 15. März beschlossen. https://www.bundestag.de/dokumente/textarchiv/2021/kw49-de-infektionsschutzgesetz-impfpraevention-870424. Zugegriffen: 25. Sept. 2022

[23] RKI (2022) Coronavirus SARS-CoV-2: Bundesbericht zu Impfquoten in Pflegeeinrichtungen – November – 2022. Datum der Berichterstellung: 04. Januar 2023. https://www.rki.de/DE/Content/Infekt/Impfen/ImpfungenAZ/COVID-19/Monatsberichte/2022-11/Bundesbericht.pdf?__blob=publicationFile. Zugegriffen: 9. Jan. 2023

[24] Gleich S, Walger P, Popp W, Lamm F, Exner M (2021) Mitteilung der Deutschen Gesellschaft für Krankenhaushygiene (DGKH). Nosokomiale COVID-19 Ausbrüche in stationären Pflegeeinrichtungen – Ursachen und Forderungen. https://www.krankenhaushygiene.de/pdfdata/2021_02_04_Ausbrueche-Pflegeeinrichtungen_HM.pdf. Zugegriffen: 22. Aug. 2022

[25] Behrendt S, Guerra FA, Räker M, Jürchott K, Klauber J, Schwinger A (2022) Die Versorgung von Pflegeheimbewohnenden am Lebensende aus Sicht der Pflege. https://www.wido.de/fileadmin/Dateien/Dokumente/Publikationen_Produkte/Buchreihen/Pflegereport/wido_pfl_befragung_lebensende_07_2022.pdf. Zugegriffen: 21. Sept. 2022

[26] Rothgang H, Wolf-Ostermann K (2020) Zur Situation der Langzeitpflege in Deutschland. Ergebnisse einer Online-Befragung in Einrichtungen der (teil)stationären und ambulanten Langzeitpflegewährend der Corona-Pandemie. https://www.uni-bremen.de/fileadmin/user_upload/fachbereiche/fb11/Aktuelles/Corona/Ergebnisbericht_Coronabefragung_Uni-Bremen_24062020.pdf. Zugegriffen: 15. Aug. 2022

[27] Dutey-Magni PF, Williams H, Jhass A (2021). COVID-19 infection and attributable mortality in UK care homes: cohort study using active surveillance and electronic records (March–June 2020). Age Ageing.

[28] Bundesregierung (2022) 10. Stellungnahme des ExpertInnenrates der Bundesregierung zu COVID-19. Zur Notwendigkeit des Infektionsschutzes für pflegebedürftige Menschen in Pflegeeinrichtungen. https://www.bundesregierung.de/breg-de/bundesregierung/bundeskanzleramt/corona-expertinnenrat-der-bundesregierung. Zugegriffen: 15. Aug. 2022

[29] Bundesgesundheitsministerium (2022) Änderung des Infektionsschutzgesetzes. https://www.bundesgesundheitsministerium.de/service/gesetze-und-verordnungen/ifsg.html. Zugegriffen: 30. Sept. 2022

[30] Hering C, Gangnus A, Budnick A (2022). Psychosocial burden and associated factors among nurses in care homes during the COVID-19 pandemic: findings from a retrospective survey in Germany. BMC Nurs.

[31] Elsbernd A, Heidecker L, Schüttemeier B (2021) Abschlussbericht Juli 2021. Studie zur aktuellen Lage in Einrichtungen der stationären und ambulanten Langzeitpflege in Baden-Württemberg (LACOVID-BA-WÜ2020). Laufzeit: 01.08.2020–31.07.2021. https://sozialministerium.baden-wuerttemberg.de/fileadmin/redaktion/m-sm/intern/downloads/Downloads_Gesundheitsschutz/Lacovid_Abschlussbericht_Elsbernd_29-07-2021.pdf. Zugegriffen: 20. Sept. 2022

[32] Yang H, Rigsby M, Zhu X, Lee C, Ory M (2022). COVID-19 in long-term care facilities: a rapid review of infection correlates and impacts on mental health and behaviors. HERD.

[33] Alonso-Lana S, Marquié M, Ruiz A (2020). Cognitive and neuropsychiatric manifestations of COVID-19 and effects on elderly individuals with dementia. Front Aging Neurosci.

[34] Wang H, Li T, Barbarino P (2020). Dementia care during COVID-19. Lancet.

[35] Deutsche Gesellschaft für Pflegewissenschaft e. V. (Hrsg) (2020) S1 Leitlinie – Soziale Teilhabe und Lebensqualität in der stationären Altenhilfe unter den Bedingungen der COVID-19-Pandemie – Langfassung – AWMF Register-nummer 184 – 001. https://register.awmf.org/assets/guidelines/184-001l_S1_Soz_Teilhabe_Lebensqualitaet_stat_Altenhilfe_Covid-19_2020-10_1-abgelaufen.pdf. Zugegriffen: 20. Sept. 2022

[36] Gangnus A, Hering C, Kohl R (2022). Covid-19-Schutzmaßnahmen und Einschränkungen des sozialen Lebens in Pflegeheimen. Pflege.

[37] AOK (2021) Heimärzte. https://www.aok-bv.de/lexikon/h/index_01189.html. Zugegriffen: 25. Sept. 2022

[38] DBfK (2019) Advanced Practice Nursing – Pflegerische Expertise für eine leistungsfähige Gesundheitsversorgung. https://www.dbfk.de/media/docs/download/Allgemein/Advanced-Practice-Nursing-Broschuere-2019.pdf. Zugegriffen: 16. Nov. 2022

[39] Iversen L, Wolf-Ostermann K, Petersen-Ewert C (2022). Welche Aufgaben hat eine Community Health Nurse? Ein Scoping Review zu Tätigkeitsfeldern am Beispiel der Versorgung von chronisch Erkrankten. Präv Gesundheitsf.

[40] Lee DT, Lee IF, Mackenzie AE, Ho RN (2002). Effects of a care protocol on care outcomes in older nursing home patients with chronic obstructive pulmonary disease. J Am Geriatr Soc.

[41] Zimmerman S, Dumond-Stryker C, Tandan M (2021). Nontraditional small house nursing homes have fewer COVID-19 cases and deaths. J Am Med Dir Assoc.

[42] Aalto UL, Pitkälä KH, Andersen-Ranberg K (2022). COVID-19 pandemic and mortality in nursing homes across USA and Europe up to October 2021. Eur Geriatr Med.

[43] Schumann H, Schmidt N (2021) Das Milliardengeschäft Altenpflege: Heime als Gewinnmaschinen für Konzerne und Investoren. Vernachlässigte Bewohner, überarbeitete Angestellte, knallharte Sparvorgaben. Und viel Geld vom Staat. Eine Recherche von Investigate Europe. https://www.tagesspiegel.de/gesellschaft/heime-als-gewinnmaschinen-fur-konzerne-und-investoren-5114386.html. Zugegriffen: 24. Sept. 2022

[44] Stall NM, Jones A, Brown KA, Rochon PA, Costa AP (2020). For-profit long-term care homes and the risk of COVID-19 outbreaks and resident deaths. CMAJ.

[45] Fried LP (2021). The need to invest in a public health system for older adults and longer lives, fit for the next pandemic. Front Public Health.

[46] Land Burgenland Zukunftsplan Pflege. Bedarfs- und Entwicklungsplanung 2018–2030. https://www.burgenland.at/fileadmin/user_upload/Bilder/Aktuelle_Meldungen/2019/Maerz/Zukunftsplan_Pflege_21_Massnahmen_fuer_die_Pflege_der_Zukunft.pdf. Zugegriffen: 12. Sept. 2022

[47] Aktiv Altern in NRW und überall (2020) Vorbildliche Altenpflege in Dänemark. https://unser-quartier.de/oberhausen/2020/07/vorbildliche-altenpflege-in-daenemark/. Zugegriffen: 17. Aug. 2022

[48] Hall K, Opitz S, Scheuermann K (2022). Die Verwaltung der Kontaktverfolgung unter COVID-19. Eine Analyse der Containment Scouts und der Mobile Tracing App.

[49] Stratil JM, Biallas RL, Burns J (2021). Non-pharmacological measures implemented in the setting oflong-term care facilities to prevent SARS-CoV-2 infections and their consequences: a rapid review. Cochrane Database Syst Rev.

